# Using Magnetoencephalography to Advance the Science of Parkinson Disease: A Systematic Review

**DOI:** 10.1002/brb3.70889

**Published:** 2025-09-21

**Authors:** Saar Kariv, Jeong Woo Choi, Mohsen Benam, Sahil Chilukuri, Amirreza Alijanpourotaghsara, Nader Pouratian

**Affiliations:** ^1^ Department of Neurological Surgery UT Southwestern Medical Center Dallas Texas USA

**Keywords:** Deep brain stimulation, Magnetoencephalography (MEG), motor network, Neurophysiology, Parkinson's disease, temporal dynamics

## Abstract

**Purpose:**

Magnetoencephalography (MEG) offers high temporal resolution and whole‐brain spatial coverage, making it a powerful tool to study brain networks. This systematic review examines the use of MEG to study Parkinson's disease (PD) brain network dysfunction, providing an update on scientific advances and identifying knowledge gaps that can be addressed using MEG as a research platform.

**Method:**

Five databases (Cochrane, Embase, PubMed, Scopus, and Web of Science) were systematically searched for MEG‐based PD publications from October 2018 (the date of the latest search in the previous systematic review) to June 2025. We used the Joanna Briggs Institute (JBI) checklist to individually assess each paper for quality and relevancy.

**Finding:**

Fifty‐four publications were grouped into motor or cognitive domains. Motor resting‐state studies were further categorized into four groups: (1) Cortical Pathophysiology and its Modulation by Therapies, (2) Basal Ganglia‐Cortical (BGC) Network Pathophysiology and its Modulation by Therapies, (3) Temporal Dynamics, Aperiodicity, and Nonlinearity in PD, and (4) Clinical Utility of MEG. We highlight increases in cortical beta power in response to L‐DOPA and its correlation with motor symptom improvement, the broad cortical distribution of beta‐gamma phase‐amplitude coupling (PAC) in PD and its link to motor symptoms, corticocortical connectivity modulation in response to different treatments, and the integration of MEG with local field potentials to study cortico‐subcortical abnormalities. Temporal dynamics, such as cortical beta bursts and reduced functional repertoire, are explored, as well as aperiodic components and nonlinearities in the power spectrum in PD. Clinically, MEG shows promise in optimizing DBS, predicting L‐DOPA response, and uncovering movement‐related and cognitive pathophysiology in PD.

**Conclusion:**

Key PD knowledge gaps that can be addressed using MEG are identified, including the need to characterize network temporal dynamics and signal interrelation in PD, the causal pathological mechanisms of the disease, and the response to different treatment modalities.

## Graphical abstract

Magnetoencephalography (MEG) offers high temporal resolution and spatial coverage, making it a powerful tool for Parkinson's disease (PD) research. This systematic review highlights recent advancements in PD research facilitated by MEG and identifies key knowledge gaps in PD research that can be addressed using MEG in the future.

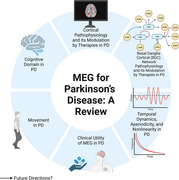

## 1 Introduction

The pathophysiology of Parkinson's disease (PD) stems from dopamine depletion in the basal ganglia, causing significant disruption of the basal ganglia‐thalamocortical circuit (BGTC) as classic parkinsonian symptoms such as bradykinesia and rigidity. Understanding BGTC dysfunction in PD can improve current therapies, guide new treatment designs, and identify critical biomarkers for disease management. Magnetoencephalography (MEG) offers excellent temporal resolution and spatial coverage and resolution and is therefore highly suitable for studying PD at the network level. A systematic review on the contribution of MEG to the study of PD was published in 2019 (Boon et al. 2019).

Since then, MEG has been used to explore more complex hypotheses about network‐level pathophysiology using novel analytical methods. Thus, an updated review is timely to synthesize the literature and provide a platform for further investigations.

Our goal in writing this review is to (1) review scientific advances that have been made in PD research and highlight how MEG uniquely advanced the science of PD, and (2) identify crucial PD knowledge gaps that can be addressed in the future by using MEG as a research platform.

We systematically review MEG‐based PD publications from October 2018 (the date of the latest search in the previous systematic review) to June 2025 (the date of the last online search). We individually assessed each paper for quality and relevance to provide an integrative update. Additionally, we propose gaps in PD research that are particularly suited to exploration using MEG.

## 2 Methods

This systematic review followed the methodology of the previous review covering literature through 2018, in accordance with the Preferred Reporting Items for Systematic Reviews and Meta‐Analyses (PRISMA) guidelines (Page et al. 2021).

**FIGURE 1:**
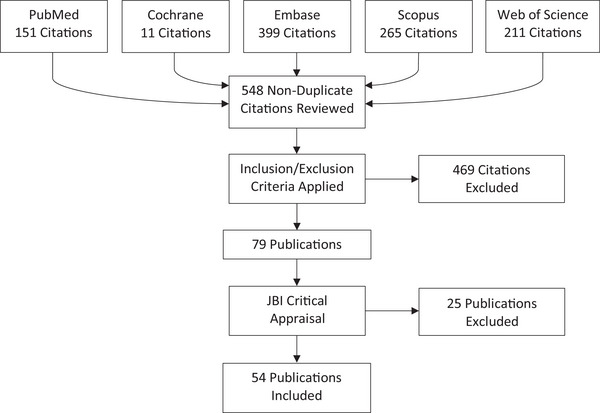
Publications inclusion flowchart.

**Search**: Five medical databases (Cochrane, Embase, PubMed, Scopus, and Web of Science) were searched with broad terms to ensure inclusiveness (see Supplementary Materials for queries). Articles published after October 15, 2018 (the date of the latest search in the previous review paper), were included, with the final search conducted on June 23, 2025. Of 1037 retrieved articles, 548 were non‐duplicates (Figure 1).

**Screening**: Titles, abstracts, and keywords were screened to include articles meeting these criteria: (a) original research, (b) English publication, (c) cohort of ≥5 subjects, and (d) MEG parameter quantification (e.g., power spectral density, coherence). Screening was performed independently by two investigators (SK, SC).

**JBI Critical Appraisal**: The Joanna Briggs Institute (JBI) checklist for case series was used, extended with an item that addresses sufficient MEG data reporting, using the same checklist as previously reported (Boon et al. 2019) (with the exclusion of sensor type and participant positioning as minimum requirements). Some publications lacked details on MEG systems (1 paper), participants' age (1 paper), or grid/resolution (2 papers) but were included for completeness. Statistical methods were not validated. Appraisal was conducted independently by two investigators (SK, MB).

**Data Collation**: A table of included publications was created (Supplementary Material Table S1), detailing key elements (e.g., title, authors, journal, cohort size, analysis space (sensor/source), and main findings). Publications were grouped into motor or cognitive domains. Within Motor, resting‐state and movement‐related studies were further categorized, with resting‐state divided into four groups:

1. Cortical Pathophysiology and Its Modulation by Therapies
2. Basal Ganglia‐Cortical (BGC) Network Pathophysiology and Its Modulation by Therapies
3. Temporal Dynamics, Aperiodicity, and Nonlinearity in PD
4. Clinical Utility of MEG

## 3 Results

### 3.1 Search and Selection

Of 548 non‐duplicate articles, 79 passed initial screening, and 54 met critical appraisal criteria. Exclusions were due to preprints/not published yet (13), missing sampling rates (3), and lack of Unified Parkinson's Disease Rating Scale Part‐III (UPDRS‐III) scores for the included participants (9).

### 3.2 Summary of Prior Review

The previous review analyzed MEG studies on PD, separating out motor network and whole‐brain approaches. In the motor network approach, early‐stage PD showed higher cortical motor beta power, while late‐stage PD had lower cortical motor beta power (Boon et al. 2019). Beta‐band corticomuscular coherence (CMC) was unchanged in early PD but decreased in later PD stages. MEG combined with local field potential (LFP) recordings employing subthalamic nucleus‐deep brain stimulation (STN‐DBS) revealed frequency‐dependent coherence between the STN and ipsilateral primary sensorimotor (S1/M1) cortex in beta and gamma bands during resting‐state. One study found increased resting‐state beta coherence between bilateral M1 in PD, which normalized with dopamine. Tremor‐associated coherence patterns between tremor harmonics and different brain areas were also identified. In the whole‐brain approach, PD progression was linked to increased power in slower bands (delta/theta) and decreased power in faster bands (beta/gamma). The review concluded that integrating motor network and whole‐brain approaches could enhance reproducibility and interpretation of findings.

### 3.3 Motor Network‐Focused Studies in PD: Resting‐State

#### 3.3.1 Cortical Pathophysiology in PD and Its Modulations by Therapies

Across the literature, there is broad agreement that beta‐band oscillations are disrupted and restored by dopaminergic treatment in PD. In advanced PD, resting motor cortical power demonstrates decreased beta oscillations compared to healthy controls (HC), which is normalized (i.e., increased) by dopaminergic medication (Boon et al. 2019). Supporting this, Cao and colleagues reported that L‐DOPA increases beta power in bilateral sensorimotor cortical areas and that the degrees of motor cortical power increase are associated with improvement in motor symptoms (akinesia and rigidity) (2020). Wiesman et al. similarly observed an increase in somatomotor beta power following dopamine replacement therapy. They also reported modulation of beta power within dopamine‐rich cortical regions, with patterns that varied across patients with PD. Notably, greater beta activity in these regions was linked to a smaller clinical response to dopamine therapy, suggesting that these changes may reflect “off‐target” effects of the medication (2025).

Beyond power changes, cross‐frequency interactions have emerged as a robust signature of PD pathophysiology. Similar to intraoperative studies (de Hemptinne et al. 2013; de Hemptinne et al. 2015; Kondylis et al. 2016), MEG has also identified cortical beta‐gamma phase amplitude coupling (PAC) as a characteristic of PD (Tanaka et al. 2022). By providing whole brain coverage and enabling comparisons to HC, MEG studies have shown that beta‐gamma PAC is distributed across sensorimotor, occipital, and temporal cortices, mirroring the spatial distribution of that seen in HC, but with significant amplification. Importantly, the akinesia severity of patients with PD is associated with the amplitude of beta‐gamma PAC only in the sensorimotor cortices (Tanaka et al. 2022). Martines et al. showed a similar pattern of spatial differences in PAC values between HC and patients with PD, which was diminished after administering L‐DOPA to the patient cohort (2023).

Compared to earlier findings that focused on localized oscillatory changes, more recent studies emphasize network‐level reorganization and compensatory connectivity, highlighting a shift in the conceptual framework of PD pathophysiology. Ambrosanio et al. employed eigenvalue centrality (EC), evaluating the influence of each node in the network on the network activity, and revealed that patients with PD showed higher frontal EC (alpha and beta bands) and lower occipital and insular EC (alpha and beta, respectively) compared to HC (2025). Frontal alpha EC in PD was associated with UPDRS‐III, indicating clinical relevance (Ambrosanio et al. 2025).

Although cortico‐cortical functional connectivity in PD is also modulated by therapeutic treatments, disagreement remains regarding the directionality of therapeutic effects on connectivity. Dopaminergic medication tends to reduce excessive phase synchrony. For instance, alpha‐band phase synchronization across MEG sensor pairs within frontal and parietal regions is elevated at baseline (aligned with Ambrosanio et al. 2025)) and suppressed by L‐DOPA (Cao et al. 2019). In addition, beta‐band phase synchrony within the parietal region (including the somatosensory area) is also suppressed by L‐DOPA (although not statistically significant), with the degree of suppression associated with the magnitude of motor symptom improvements (UPDRS‐III total score) (Cao et al. 2019). On the other hand, DBS increases high‐beta/low‐gamma cortico‐cortical phase synchrony in motor, occipitoparietal, middle temporal, and prefrontal cortical regions, enhancing network efficiency and modularity (Wang et al. 2022). This contrast underscores a divergence in the network‐level mechanisms of pharmacological versus DBS treatments. Supporting this, Boon et al. found DBS‐induced connectivity changes in high‐alpha/low‐beta bands, which are increased in the frontobasal areas and reduced in other more dispersed regions (2020). Notably, motor symptom improvement (total UPDRS‐III, bradykinesia, and rigidity) was associated with low‐beta connectivity increase in the sensorimotor cortex, whole cortex, and subcortical regions (Boon et al. 2020).

Another neuromodulatory intervention, MR‐guided focused ultrasound (MRgFUS), has demonstrated region‐specific changes in connectivity. Visani et al. found that tremor alleviation following MRgFUS (targeting the ventralis intermedius nucleus) was associated with increased cortico‐muscular coherence and decreased long‐range corticocortical beta connectivity, suggesting that symptom relief may emerge from both enhancement and suppression of different network components (2024).

Finally, taking a neurochemical perspective, Wiesman et al. found that broadband (across multiple frequency bands) resting‐state power alterations (when compared to HC) occur mainly in regions rich in acetylcholinergic, glutamatergic, and serotonergic systems (2024). Interestingly, stronger co‐localization between these neurophysiological alterations and neurochemical maps was linked to better cognitive function, suggesting a complex interplay between neurochemistry and brain activity in PD (Wiesman et al. 2024).

Taken together, these findings suggest emerging areas of consensus—such as the pathological amplification of cortical beta synchrony and PAC, and the modulating effects of dopaminergic and DBS treatments–alongside points of divergence regarding treatment mechanisms and spatial patterns of connectivity. A clearer mechanistic understanding of these differences will likely require integration across modalities and symptom domains (see knowledge gap 4 in the discussion section).

#### 3.3.2 BGC Pathophysiology in PD

Given that PD involves impairment of the BGC network, simultaneous recordings of LFP (from externalized DBS lead in STN or globus pallidus internus (GPi)) and MEG (from cortices) have been a powerful multimodal platform to explore how STN/GPi and different brain regions (both cortical and subcortical) are interrelated. Along with the advanced neuroimaging techniques, more recent works have focused on the spatial and functional mapping within BGC networks in PD. For instance, van Wijk et al. reported that STN‐brain coherence topologies are frequency dependent such that STN theta/alpha oscillations are most coherent with the thalamus, while STN beta oscillations are most coherent with the premotor cortex. Although considerable spatial overlap is observed within the STN, STN contacts showing theta/alpha‐dominant coherence are located significantly more inferiorly (towards the limbic part of the STN) than STN contacts showing beta‐dominant coherence (2022).

Oswal and colleagues utilized multimodal neuroimaging methods, including STN and GPi LFP and MEG, as well as MRI‐based tractography, to investigate the relationship between cortico‐subcortical functional and structural connections in PD (2021). They found greater cortical coherence with STN compared to GPi, especially at the high‐beta rather than low‐beta range, and that this high‐beta coherence is clustered at the mesial M1 and supplementary motor area (SMA). Using Granger causality analysis, they showed that the SMA/mesial M1 drives the STN in the high‐beta range. Interestingly, the high‐beta SMA‐STN coherence is associated with voxel‐wise fiber density between these structures (presumably representing the “hyper‐direct pathway”), highlighting the propagation of the cortical high‐beta oscillations to STN via the hyper‐direct pathway (Oswal et al. 2021). Using these MEG‐derived results, the authors used computational modeling to suggest that prominent high‐beta “hyper‐direct” activity may generate subcortical pathological activity at the low‐beta range.

#### 3.3.3 Temporal Dynamics, Aperiodicity, and Nonlinearity in PD

MEG's high spatial and temporal resolution enables advanced, multidimensional analyses that go beyond traditional measures like spectral power and coherence. In this section, we categorize the pathophysiological findings, focusing on (1) temporal dynamics of abnormal oscillations, (2) aperiodic components of neural signals, (3) nonlinear characteristics of signals, and (4) temporal dynamics of topological characteristics.

##### 3.3.3.1 Temporal Dynamics of Abnormal Oscillations

While band‐specific power is traditionally analyzed by averaging spectral power across relatively long periods of up to several minutes, there has been increased interest and attention to the temporal dynamics of these spectral changes, with a focus on transient changes in spectral power. Transient increases in beta power are commonly called “beta bursts” and can be quantitatively characterized by exploring the duration and amplitude of each burst and the burst rate (e.g., burst occurrences per second). These beta burst characteristics have shown promise as MEG‐based biomarkers for PD (Pauls et al. 2022; Vinding et al. 2020). For example, patients with PD have a lower beta burst rate in the sensorimotor cortices compared to HC, which is correlated with the severity of motor symptoms (bradykinesia and tremor) (Vinding et al. 2020; Vinding et al. 2024). More recently, larger and longer beta bursts in the sensorimotor cortex have been found in patients with PD compared to HC, which were found to be normalized by STN‐DBS (Pauls et al. 2022). The degree of reductions of beta burst amplitude is associated with improvements in motor symptoms (Pauls et al. 2022).

Changes in subcortical beta burst patterns are also considered to reflect PD pathophysiology, with longer beta bursts in STN being correlated with greater motor impairments and beta bursts duration being shortened by dopaminergic medication (Tinkhauser et al. 2017). Sure and colleagues investigated the relationship between STN beta burst and MEG‐based cortical activity by exploring the cortical event‐related magnetic field (ERF) time‐locked to STN bursts (2022). ERF activity was not restricted to motor cortical regions but occurred in multiple cortical areas, including motor, limbic, and associative, as well as areas involved in visual and language processing, indicating the involvement of STN beta bursts in the formation of multiple STN‐cortical loops in PD (Sure et al. 2022).

Temporal dynamics in contexts other than beta bursts have also been studied: recent works have shown that an individual's neuroimaging phenotype remains surprisingly stable over time and thus can be considered a physiological “fingerprint” (Finn et al. 2015). Da Silva Castanheira et al. used resting‐state MEG to examine these patterns in PD and their temporal dynamics (2024). Patients with PD showed lower identification accuracy (81.1% vs. 89.8% in HC) and higher moment‐to‐moment spectral variability, reducing self‐similarity over time. However, when isolating only the rhythmic components (by projecting out the arrhythmic 1/f component), patients with PD showed lower variability than controls (da Silva Castanheira et al. 2024).

##### 3.3.3.2 Aperiodic Characteristics of Neural Activity

Besides oscillations, the aperiodic part of neural population activity can also provide insights into network dysfunction in PD, given that it is presumed to be related to the balance between excitatory and inhibitory synaptic currents (Donoghue et al. 2020). Using MEG, Helson and colleagues calculated the aperiodic component of the power spectrum of neural activity by fitting a power law (i.e., κ/f λ, where f is the frequency, and κ and λ are the fitting parameters) (2023). In all but frontal regions, λ is significantly larger in PD than in HC. Interestingly, dopaminergic medications do not change λ. Temporally, λ is not constant across time, and a comparison of this variation between PD and HC revealed that λ is significantly less variable in PD compared to HC across multiple brain regions. Taken together, these MEG studies demonstrated that non‐frontal neural activity in patients with PD is “slower” and temporally less variable compared to HC. Of note, in patients with PD, λ was positively correlated with age but not with UPDRS‐III (Helson et al. 2023). Diving deeper into the subject, Wiesman et al. found a topographical relationship between the cortical “slowing” (of aperiodic and periodic components) in patients with PD and clinical (motor and cognitive) impairment along the sagittal plane, such that “posterior” slowing is associated with worse clinical impairment (2023). In contrast, “anterior” slowing was associated with less clinical impairment and thus may indicate a compensatory mechanism (Wiesman et al. 2023).

##### 3.3.3.3 Nonlinear Components of Neural Signals

While the traditional approaches of analyzing oscillatory activity, such as spectral power and coherence, have been successful in revealing PD pathophysiology, those measures depend on linear characteristics of the underlying signals. However, the brain is known to be a nonlinear and complex system deriving nonlinear and nonstationary signals, which has been underexplored in PD. Özkurt and colleagues hypothesized that STN time series in patients with PD contain nonlinear components that reflect abnormal information load and that dopaminergic medication diminishes this effect (2020). They applied a “nonlinearity” metric (nonlinear short‐time average magnitude difference function, or “nsAMDF”; see (Özkurt et al. 2020) for the details) to measure nonlinearity in STN LFPs and MEG signals. For STN, the nonlinearity measure in the high‐beta range is significantly higher in the off‐medication state compared to the medicated state and is significantly correlated with contralateral tremor score. Conversely, nonlinearity measures from cortical sources in the alpha‐band range are significantly higher and correlate with contralateral akinesia score in the medicated state compared to the unmedicated state.

##### 3.3.3.4 Temporal Dynamics of Topological Characteristics

Normal brain functions are known to employ flexible dynamics of different brain networks. Thus, the temporal properties of specific networks, such as overall temporal presence, the interval between different networks, and the number of networks, can provide unique insights to understand brain function, especially assuming that their alterations might be associated with neurological disorders like PD (Sharma et al. 2021; Sorrentino et al. 2021).

One study characterized the effect of dopaminergic medication on oscillatory brain networks in PD, employing a hidden Markov model (HMM) on simultaneous STN LFP and MEG recordings (Sharma et al. 2021). HMM is a data‐driven algorithm that finds recurrent patterns in multivariate time series based on probabilistic calculations. Here, HMM analysis was able to separate key networks such as cortico‐STN and STN‐STN networks. Dopaminergic medications modulate beta‐band cortico‐STN network topology from an STN‐mediated motor network to a frontoparietal‐mediated one, implying a reduction of communication between STN and cortex. On the contrary, local STN‐STN coherence is not modulated by medication (Sharma et al. 2021).

Similarly, Wei et al. used HMM to identify four cortical activation states in PD, including a motor state (2025). Both L‐DOPA and DBS modulated beta activity in this state, but in opposite ways: L‐DOPA increased low and high beta activity, while DBS decreased high beta activity (2025). These findings, together with those of Cao and Wang of different connectivity modulation patterns, suggest that L‐DOPA and DBS have distinct therapeutic mechanisms (Cao et al. 2019; Wang et al. 2022). Sorrentino and colleagues studied the “functional repertoire” in PD, which is defined as the number of distinct topographical patterns of cortical source activations over time, reflecting the degree of flexibility in the brain (e.g., a smaller repertoire represents less flexibility) (2021). Patients with PD have less “functional repertoire” compared to HC, which is thought to point toward impaired brain flexibility. Interestingly, the size of the functional repertoire in patients with PD correlates negatively with both global corticocortical phase synchronization in the beta band and UPDRS‐III scores. Beta‐band hyper‐synchronization across the network in PD is thought to reduce the degree of freedom and brain flexibility, thereby interfering with normal motor functions (Sorrentino et al. 2021).

#### 3.3.4 Clinical Utility of MEG

MEG is not routinely used for assessing PD, but its fast, comfortable whole‐brain imaging has potential clinical value, especially for patients with DBS. Spooner and colleagues provided evidence suggesting that MEG may have clinical utility in optimizing DBS parameters in patients with PD (2023). They hypothesized that optimal (clinically effective) orientations of directional DBS contacts would elicit a larger sensorimotor evoked response to stimulation and that the larger sensorimotor evoked response would predict clinical performance. Patients with PD were stimulated with STN‐DBS in different contact orientations (optimal, anterior, medial, lateral, and omnidirectional). For each contact orientation, sensorimotor evoked response to single‐pulse stimulation was also separately recorded. Optimal contact orientations yielded overall better performance of finger tapping and produced stronger evoked responses in the sensorimotor cortex when compared to other non‐optimal contact orientations. Finally, a stronger sensorimotor evoked response was shown to be able to predict smoother finger‐tapping movements.

Another study used MEG and LFP recordings with machine learning to predict STN‐DBS efficacy (Hirschmann et al. 2022). While both STN power and STN‐cortical coherence predicted DBS outcomes, coherence‐based features were more efficient in doing so. High‐beta power was the important STN local feature, whereas STN‐parietal coherence in low‐gamma (36–60 Hz) and high‐frequency oscillations (> 200 Hz) played a dominant role in coherence‐based predictions.

Traditionally, PD research (and perhaps more collectively—brain research) is performed by averaging data across all subjects per group and, in turn, performing the group‐level comparison. Pivoting away from this approach, Peña et al. used motor‐cortical MEG power analysis to investigate the subject‐specific response to L‐DOPA and whether this response can predict motor improvement (2021). In this analysis, 17 patients with PD were recorded before and after administration of short‐duration L‐DOPA, and a linear support vector machine (SVM) was constructed per subject to distinguish between L‐DOPA‐ON and L‐DOPA‐OFF. Clinically, short‐duration L‐DOPA significantly improved most of the UPDRS‐III motor scores. SVM's ability to classify between medication states was variable across subjects, but classification accuracies were constantly above chance (i.e., > 0.5) in 10 out of 17 patients with PD. Next, per‐subject, canonical‐frequency band weights were constructed based on each band's contribution to the classification accuracy. These frequency band weights exhibited marked inter‐subject variability but, interestingly, relatively low intra‐subject variability (reaching consistency in 13 out of 17 participants)—implying the motor cortical brain response to L‐DOPA may be unique in each patient. Lastly, there was a significant correlation between subject‐specific left upper extremity improvement in bradykinesia and classification accuracy (this correlation did not survive multiple comparison corrections).

### 3.4 Movement‐related Changes in Brain Activity in PD

Oscillatory dysfunction in patients with PD can also be studied in the context of movement, which allows better characterization of abnormalities of the motor network in preparation for and during actual movement execution.

#### 3.4.1 Movement Preparation

Alterations in movement preparation have been increasingly linked to aberrant beta oscillatory dynamics in PD, particularly with respect to pre‐stimulus beta suppression. In a motor sequence learning task, Meissner et al. found that patients with PD showed impaired learning—as indexed by reduced reaction time (RT) benefits in sequence versus random blocks—and exhibited diminished pre‐stimulus motor cortical beta suppression. Critically, the degree of this suppression predicted learning performance, suggesting that beta modulation plays a direct role in motor preparation and behavioral adaptation (2019).

While pre‐movement suppression of beta power in motor cortices is a well‐recognized phenomenon, the mechanism by which it occurs is less clear. Heideman et al. (2020) used analyzed beta event dynamics during movement preparations in the cued Go/NoGo task, incorporating valid and invalid temporal cues. Beta event dynamics analysis was performed on the sensor‐level data using HMM analysis that was used to label each time point as being in an “on” or “off” beta state (analogous to having high or low momentary beta power in traditional analysis approaches). The HC group showed a higher anticipation behavior, reflected by a larger difference in RT between valid cue trials (early target after expecting early cue) and invalid cue trials (early target after expecting late cue). HMM showed that the general decrease in beta power during the anticipatory period in both groups arises from an increased interval between beta events (= “beta bursts”), not due to other factors such as a decrease in beta burst amplitude/duration, and that this modulatory mechanism for beta suppression is less prominent in patients with PD than in HC. Finally, a significant correlation was found in the HC group between behavioral performance and increase in interval time between beta bursts in valid versus invalid cues, implying that beta burst modulation capacity is associated with improved behavioral performance.

Further extending the scope of preparatory dysfunction in PD, Waldthaler et al. explored the cortical dynamics underlying response inhibition during the mental preparation for the anti‐saccade task, in which participants are instructed to suppress a reflexive eye movement in the direction of a presented stimulus and to execute a volitional eye movement in the opposite direction as fast as possible (2022). Anti‐saccade latencies, which are defined as the time from stimulus onset to the start of the first saccade, were significantly higher in the PD group compared to HC, while the rate of directional errors was not significantly different between groups. During the anti‐saccade preparation period, the HC group had a significant increase in right dorsolateral prefrontal cortex (DLPFC) beta power, while the PD group did not. Concurrently, alpha and lower beta band powers in the bilateral frontal eye fields (FEFs) were significantly lower in the PD group than in HC. The intensity of this reduction was found to correlate with the anti‐saccade latency in both groups, so that stronger power reduction was significantly associated with longer anti‐saccade latency. Finally, connectivity analysis revealed reduced alpha band connectivity between the right DLPFC and right superior FEF in the PD group compared to HC (Waldthaler et al. 2022). Taken together, these findings suggest that alteration in the right DLPFC hinders proactive response inhibition in the form of impaired “top‐down” cortical inhibition and thus increases anti‐saccade latencies in PD.

Overall, across diverse movement paradigms—from motor sequence learning to response anticipation and inhibition—studies consistently show that PD is associated with reduced capacity to flexibly modulate beta activity during movement preparation, whether via insufficient burst suppression (Meissner et al., Heideman et al.) or inadequate top‐down engagement of control regions (Waldthaler et al.). While beta suppression has been broadly linked to movement readiness, these findings reveal distinct mechanisms—from sensorimotor beta burst dynamics to prefrontal network connectivity—that may underlie specific facets of preparatory dysfunction in PD.

#### 3.4.2 Movement Execution

Altered cortical and subcortical dynamics during movement execution have been widely observed in PD, often implicating impaired beta‐band modulation as a central feature. Disbrow et al. used MEG to examine event‐related changes to a cue/target task that required initiating uncued movement (activation) or inhibiting cued movement (inhibition) in HC and patients with PD (2022). The activation inhibition tasks consisted of a cue arrow followed by a target arrow indicating the response to be produced. A Unidirectional arrow indicated a unimanual button press, and a bidirectional arrow indicated a bimanual button press. There were three types of trials: matched, where the cue arrow and target arrow are equal; activation, where subjects were required to activate an uncued button press (for e.g., cue: right arrow, target: bilateral arrow); and inhibition where subjects were required to inhibit a cued button press (for e.g., cue: bilateral arrow, target: right arrow). While reaction time was not significantly different between groups, patients with PD had an increased error rate compared to HC. The following brain regions showed statistically significant event‐related changes in oscillatory power during movement (following response): BA 4 (M1), lateral BA 6 (preSMA), medial BA 6 (SMA), BA 7 (posterior parietal cortex), BA 24 (anterior cingulate cortex), and BA 9/10 (medial/anterior prefrontal cortex). Compared to HC, the PD group showed significant increases in beta power in bilateral M1 and a concurrent decrease in the alpha and beta power in the contralateral SMA and the medial/anterior prefrontal cortices, respectively. Patients with PD also showed a significantly higher peak latency in these areas for different frequency bands. Thus, reduced behavioral performance may be explained by aberrant brain activity during movement, specifically in brain regions responsible for activation and inhibition.

Vinding et al. studied differences in cortical processing of a passive movement between PD participants and HC (2019). Proprioceptive information consisting of controlled passive movement of the index finger was analyzed and compared between PD med off, PD med on, and HC. Sensor‐level time‐frequency analysis showed a significantly attenuated post‐movement beta rebound (PMBR) in the PD group compared to HC. Interestingly, there was no significant difference in the time‐frequency response between medication on and off in PD. These results demonstrate a disease‐related deterioration in cortical processing of proprioceptive afference in PD, which seems to be unaffected by medications.

Winkler et al. combined STN LFP and MEG to study how beta synchronization in PD varies with movement type and expectation (2024). Participants were instructed to start, stop, or reverse (i.e., change the direction of) a wheel with their index fingers, according to a visual stimulus. Movement starts triggered beta suppression, and movement stops showing beta rebound in both the STN and the motor cortex. Movement reversals caused cortical beta suppression but no rebound. A decrease in beta cortico‐STN coherence, with a concurrent increase in gamma cortico‐STN coherence, was observed immediately following movement start, and an increase in beta coherence (“rebound” beta coherence) was observed immediately after movement stops. When comparing predictable versus unpredictable conditions, beta power suppression at movement start was stronger in the predictable conditions, while beta coherence “rebound” at movement stop was stronger in unpredictable conditions. The latter implies a stronger event‐related interaction between STN and cortex in unpredictable conditions (Winkler et al. 2024). Cao et al. further dissected the contribution of beta cortico‐subcortical coherence to movement initiation and inhibition by separately studying low beta and high beta coherence in a classical go/no‐go task (2024). Using MEG and GPi/STN LFPs, they found low beta coherence focus in the ipsilateral primary sensorimotor cortex (referred to as “lateral” focus) and high beta coherence focus at a more medial and frontal location near the SMA (“medial” focus). Similar to Winkler et al. (2024), low beta coherence decreased during go trials and rebounded after response. High beta coherence, on the other hand, showed a more complex pattern—a significant increase in the unpredicted nogo trials compared to predicted nogo trials, at a relatively long latency of 3 s post‐stimulus (Cao et al. 2024).

Taken together, across active and passive movement paradigms, the recent studies reveal a converging dysfunction in beta oscillatory and coherence dynamics during movement execution in PD. They include amplified or poorly timed beta synchronization, attenuated PMBR, and context‐sensitive disruptions in cortico‐subcortical coherence, which is considered a failure of the network communications to adaptively modulate with task demands. The fact that some of these deficits (e.g., PMBR attenuation) are insensitive to dopaminergic medication further supports the idea that multiple, partially distinct mechanisms—involving both motor output and sensory feedback systems—contribute to impaired motor execution in PD.

### 3.5 Utilizing MEG for Cognitive Research in PD

Despite being called a movement disorder, non‐motor impairments are well recognized in PD. MEG technology helps study brain networks in cognitively impaired patients with PD, develop biomarkers, and differentiate PD‐related cognitive impairment from other diseases.

Rucco et al. compared resting‐state brain networks in HC, PD with normal cognition (PD‐NC), and PD with cognitive impairment (PD‐CI) (2022). They evaluated global and local functional connectivity and applied a minimum spanning tree (MST, a set of connections that joins all nodes in the graph into a single contiguous tree with minimum possible total connection distance) algorithm to describe the network from a graph analytic perspective. They found reduced global and local connectivity in several brain areas and large‐scale network rearrangements in PD‐CI patients, compared to HC and PD‐NC patients. For example, the PD‐CI group has a significantly elevated fraction of nodes connected to the network by only one connection (leaf fraction) compared to the other groups. Interestingly, the Montreal Cognitive Assessment (MoCA) score was associated with some of the graph analytic measures, such as the diameter, defined as the network's longest‐shortest path.

Similarly, Simon et al. used graphtheory to assess connectivity differences in PD‐NC, PD with mild cognitive impairment (PD‐MCI), and PD with dementia (PDD) (2021). They applied MST analysis, revealing distinct connectivity patterns between the groups that may aid clinical assessment. Simon et al. also confirmed that “spectral slowing” is linked to cognitive decline in PD, most notably as PD‐NC progresses to MCI (Simon et al. 2022). Hyder et al. used MEG to analyze spoken language processing in early‐stage PD versus HC, achieving 78% classification accuracy using functional connectivity and logistic regression, highlighting MEG sensitivity to subtle neurological changes (2021).

Shifting focus, Boon et al. explored apathy in DBS‐treated patients with PD (2021). In 26 patients with PD, apathy scores were recorded before and > 6 months after STN DBS. Overall, apathy severity significantly increased after DBS. Apathy change correlated positively with more dorsolateral stimulation of the left STN and negatively with alpha1 (8‐10 Hz) global functional connectivity of the DLPFC. Apathy change was not correlated with dose reduction of dopaminergic medication. These observations, together with previous studies showing that functional changes in the DLPFC are associated with cognitive apathy, suggest that apathy increase in DBS‐treated patients may be related to the stimulation itself (Boon et al. 2021).

## 4 Discussion

This review systematically examines the use of MEG in studying PD. We highlight cortical beta power's increase in response to L‐DOPA and its correlation with motor symptom improvement, along with beta‐gamma PAC broad cortical distribution in PD and its link to motor symptoms. We discuss corticocortical connectivity changes—decreasing with L‐DOPA and increasing with DBS—and the integration of MEG with LFPs, revealing a correlation between high beta SMA‐STN coherence and “hyper‐direct” pathway fiber density. Temporal dynamics, such as cortical beta bursts and reduced functional repertoire, are explored, as well as distinctive power spectrum aperiodic components in PD. Nonlinear STN high‐beta activity, which is elevated OFF medication and correlated with symptoms, suggests its potential as a disease marker. Clinically, MEG shows promise in optimizing DBS, predicting L‐DOPA response, and uncovering movement‐related and cognitive pathophysiology in PD. Despite these advancements, key knowledge gaps in the study of PD remain and can be addressed by using MEG:

**Knowledge gap 1** – Dynamic interrelation of network activity in PD

As highlighted above, recent studies successfully revealed alterations in cortical oscillatory activities underlying PD pathophysiology. For example, local beta power, local PAC, and cortico‐cortical coupling were found to be altered in PD when compared to HC (Boon et al. 2020; Cao et al. 2020; Cao et al. 2019; Tanaka et al. 2022; Wang et al. 2022). While each finding is interesting in and of itself, ultimately we should seek to understand the interrelationship between these seemingly distinct signals and physiological findings. Oswal et al. (2021) suggested the high beta oscillations from the cortex drive STN oscillations, which may then subcortically propagate in the form of pathological low beta activity. A similar cascadic representation that accounts for a broader range of pathological events would help establish a more integrated understanding of PD pathophysiology and is therefore necessary.

**Knowledge gap 2** – Causal pathological mechanisms in PD

Beyond understanding network‐level interactions, there is a clear gap in understanding causal pathophysiological mechanisms. Which of the established PD biomarkers are critically pathological? In contrast, which physiological events are not directly linked to symptomatology? A sophisticated approach to answering this question would be to utilize established therapeutic interventions to study therapy‐dependent modulation of distinct PD biomarkers. For example, one can introduce an effective vs. ineffective DBS pattern and compare the differential modulation of the two. This would enable the characterization of therapeutic vs. nonspecific stimulation‐dependent modulation, helping to establish a causal relationship between biomarkers and symptoms. Describing PD pathophysiology in a single network model is important for understanding causal relationships. For example, patients with PD have a smaller number of cortical activation patterns compared to HC, suggesting less brain flexibility (Sorrentino et al. 2021). Can this observation be explained in the context of the criticality and complexity of operating systems? An increasing number of scientists are adopting the hypothesis that the brain is a complex system operating at a specific criticality state (Haimovici et al. 2013). The criticality and information theory approach suggests that neural networks at or near critical states exhibit optimal information transmission, information storage, and computational power. In PD, the neural network may be positioned away from the critical and towards a hyper‐synchronized state, thereby inducing less efficient communication (West et al. 2016) and reduced brain flexibility (Sorrentino et al. 2021). Such an inclusive model can act as common ground for further studies and expedite the development of novel therapeutic approaches.

**Knowledge gap 3** – Temporal dynamics and moment‐to‐moment correlation of cortical oscillations and behavior (especially kinematics) in PD

Despite the dynamic and non‐stationary nature of neural oscillations, the relative contribution of temporally dynamic changes in neural oscillations to PD symptomatology remains underexplored. In fact, we suggest that the non‐stationary nature of the neural activity has likely contributed significantly to discrepancies in previous PD studies. How do beta bursts relate in time and space, and how do these inter‐regional relationships relate to PD symptoms? Furthermore, how do these oscillatory transient bursts originate in the brain? (O'Keeffe et al. 2020). The moment‐to‐moment relationship between cortical oscillations and kinematics in PD likewise remains underexplored. Finally, movement modulates many neurophysiological signals in a similar manner as therapeutic interventions for PD (for example, reduced M1 beta power or basal ganglia‐cortical beta coupling) (AuYong et al. 2018; Choi et al. 2020; Wang et al. 2018). Yet, most studies separately analyze the effect of movement and therapy on BGC physiology. Simultaneous assessment of cortical oscillations and kinematics is critical to identify novel biomarkers during activation of the motor network.

**Knowledge gap 4** – Modulation of PD neurophysiology by therapy

Unlike STN/GPi, there is no consensus on how cortical oscillations are modulated by different therapeutic modalities. Different patterns of cortical modulation have been observed in response to dopaminergic medications and DBS: medication decreases corticocortical coupling in alpha/beta (Cao et al. 2019), while DBS increases it in a partially overlapping frequency range (Boon et al. 2020; Wang et al. 2022). Understanding differential effects of distinct therapies at a brain‐wide level will enable further dissection and understanding of basal ganglia thalamocortical network pathophysiology in PD, not only illuminating the physiologic basis of disease but also providing greater insight into therapeutic mechanisms of distinct interventions.

### 4.1 Limitations of MEG

While MEG has enabled significant advances in PD research, several limitations should be considered:

1. Spatial resolution and inverse problem: MEG detects brain signals that are 10–100 million times weaker than Earth's magnetic field (Baillet 2017). It is also less sensitive to radial currents (Baillet et al. 2001) and relies on solving an “ill‐posed” inverse problem with no unique solution, requiring modeling assumptions (Baillet 2017). These factors can reduce spatial accuracy. Still, the literature demonstrates that MEG has substantially better spatial localization compared to EEG (Leahy et al. 1998).
2. Sensitivity to deep brain structures: MEG's signal strength decreases with depth, limiting detection of subcortical activity (Baillet 2017)—which is very relevant in PD. While advanced signal processing (Samuelsson et al. 2019) or combining MEG with deep structures LFP recordings can help, the latter is invasive, limited in coverage, and technically complex.
3. Cost and infrastructure: Traditional MEG systems are expensive and have high maintenance costs due to superconducting Quantum Interference Devices (SQUID)‐based sensors requiring liquid helium to support cryogenic cooling (of temperature close to absolute zero (Gross 2019)) and operation in magnetically shielded rooms (Baillet 2017). However, newer technologies like optically pumped magnetometers (OPMs) do not require liquid helium and show promise of significantly reduced costs (Savukov and Romalis 2005).
4. Head position dependence and system stationarity: MEG signal quality is affected by head movement (Baillet 2017)—an issue for patients with PD. Modern systems offers real‐time head tracking, but movement correction adds complexity. Implants like DBS may also interfere with tracking systems. Additionally, traditional MEG systems require a fixed position, which can be difficult for patients with kyphosis or rigidity associated with PD. Future OPM systems may help with these problems as well. Finally, unlike EEG, MEG systems are currently stationary, which limits the behaviors that can be directly probed (for example, walking cannot be examined with MEG due to its lack of mobility).

## 5 Conclusion

This systematic review highlighted recent advancements in PD research using MEG and identified key knowledge gaps for future investigation. As brain science evolves, MEG technology—with its high temporal resolution and spatial coverage—can serve as an essential tool for deepening our understanding of PD pathophysiology through advanced analytical methods. Future MEG systems will reduce some of the technology limitations, thereby enabling greater availability and flexibility.

## Author Contributions

**Saar Kariv**: data curation, formal analysis, methodology, writing – original draft, writing – review and editing. **Jeong Woo Choi**: formal analysis, investigation, writing – original draft, writing – review and editing. **Mohsen Benam**: formal analysis, writing – original draft. **Sahil Chilukuri**: data curation, formal analysis, writing – original draft. **Amirreza Alijanpourotaghsara**: formal analysis, writing – original draft. **Nader Pouratian**: conceptualization, funding acquisition, project administration, supervision, writing – review and editing

## Ethics Statement

Ethical approval was not required for this literature review.

## Conflicts of Interest

The authors declare no conflicts of interest.

## Peer Review

The peer review history for this article is available at https://publons.com/publon/10.1002/brb3.70889

## Supporting information




**Supplementary Material**: brb370889‐sup‐0001‐SuppMatt.docx

**Supplementary Table**: brb370889‐sup‐0002‐TableS1.docx

## Data Availability

Data sharing is not applicable to this article as no new data were created or analyzed in this study.
