# Supplementary material for: Using Magnetoencephalography to Advance the Science of Parkinson Disease: A Systematic Review
Source: Brain Behav. 2025 Sep 21;15(9):e70889. doi: 10.1002/brb3.70889 (PMC12451052; doi:10.1002/brb3.70889)
Supplement: Supplementary file 1 — Supplementary Material: brb370889‐sup‐0001‐SuppMatt.docx [file BRB3-15-e70889-s002.docx]

**CHOCHRANE:**

Advanced search: search: title abstract keyword:

((parkinson) or (parkinson disease) or (parkinson's disease) or (idiopathic parkinson's disease) or (parkinson's disease, idiopathic) or (parkinson disease, idiopathic) or (primary parkinsonism) or (parkinsonism, primary) or (paralysis agitans)) AND ((magnetoencephalography) or (magneto-encephalography) or (magnetoencephalogram) or (magneto-encephalogram) or (magnetoencephalograms) or (magneto-encephalograms) or (magnetoencephalogr*) or (magneto-encephalogr*) or (meg))

**EMBASE:**

1 parkinson.af. 216555

2 parkinson disease.af. 195173

3 parkinson's disease.af. 170284

4 idiopathic parkinson's disease.af. 4848

5 parkinson's disease, idiopathic.af. 17

6 parkinson disease, idiopathic.af. 4

7 primary parkinsonism.af. 44

8 parkinsonism, primary.af. 7

9 paralysis agitans.af. 155

10 magnetoencephalography.af. 15640

11 magneto-encephalography.af. 246

12 magnetoencephalogram.af. 481

13 magneto-encephalogram.af. 23

14 magnetoencephalograms.af. 68

15 magneto-encephalograms.af. 1

16 magnetoencephalogr*.af. 16085

17 magneto-encephalogr*.af. 330

18 meg.af. 20301

19 1 or 2 or 3 or 4 or 5 or 6 or 7 or 8 or 9 244934

20 10 or 11 or 12 or 13 or 14 or 15 or 16 or 17 or 18 26809

21 19 and 20 612

**PUBMED:**

Advanced 🡪 all fields
Search: **((parkinson) or (parkinson disease) or (parkinson's disease) or (idiopathic parkinson's disease) or (parkinson's disease, idiopathic) or (parkinson disease, idiopathic) or (primary parkinsonism) or (parkinsonism, primary) or (paralysis agitans)) AND ((magnetoencephalography) or (magneto-encephalography) or (magnetoencephalogram) or (magneto-encephalogram) or (magnetoencephalograms) or (magneto-encephalograms) or (magnetoencephalogr*) or (magneto-encephalogr*) or (meg))** Sort by: **Most Recent**

**SCOPUS:**

TITLE-ABS-KEY ( ( ( parkinson ) OR ( parkinson AND disease ) OR ( parkinson's AND disease ) OR ( idiopathic AND parkinson's AND disease ) OR ( parkinson's AND disease, AND idiopathic ) OR ( parkinson AND disease, AND idiopathic ) OR ( primary AND parkinsonism ) OR ( parkinsonism, AND primary ) OR ( paralysis AND agitans ) ) AND ( ( magnetoencephalography ) OR ( magneto-encephalography ) OR ( magnetoencephalogram ) OR ( magneto-encephalogram ) OR ( magnetoencephalograms ) OR ( magneto-encephalograms ) OR ( magnetoencephalogr* ) OR ( magneto-encephalogr* ) OR ( meg ) ) )

**WEB OF SCIENCE:**

Advanced search: all fields:

**ALL=(((parkinson) or (parkinson disease) or (parkinson's disease) or (idiopathic parkinson's disease) or (parkinson's disease, idiopathic) or (parkinson disease, idiopathic) or (primary parkinsonism) or (parkinsonism, primary) or (paralysis agitans)) AND ((magnetoencephalography) or (magneto-encephalography) or (magnetoencephalogram) or (magneto-encephalogram) or (magnetoencephalograms) or (magneto-encephalograms) or (magnetoencephalogr*) or (magneto-encephalogr*) or (meg)))**
