# Supplementary material for: Using Magnetoencephalography to Advance the Science of Parkinson Disease: A Systematic Review
Source: Brain Behav. 2025 Sep 21;15(9):e70889. doi: 10.1002/brb3.70889 (PMC12451052; doi:10.1002/brb3.70889)
Supplement: Supplementary file 2 — Supplementary Table: brb370889‐sup‐0002‐TableS1.docx [file BRB3-15-e70889-s001.docx]

**Table 1 – Included Articles**

| **Publication name** | **1^st^ author/corr. author** | **Year** | **Journal** | **Center (corr. author affil.)** | **N** | **JBI** | **Source/sensor space** | **Main findings** |
| --- | --- | --- | --- | --- | --- | --- | --- | --- |
| The effect of L-dopa and DBS on cortical oscillations in Parkinson's disease analyzed by hidden Markov model algorithm | Kunzhou Wei / Xiangyan Kong or Chunyan Cao | 2025 | Neuroimage | Department of Neurosurgery, Affiliated Ruijin Hospital, Shanghai Jiao Tong University School of Medicine, Shanghai, China | PD=21(L-DOPA), 11 (DBS) | 6 | source | **Main point: L-DOPA and STN DBS induce different effects on cortical beta activity in patients with PD.**   - Resting state MEG analyzed in patients with PD by implementing a hidden Markov model (HMM) to analyze cortical spatiotemporal patterns and their modulation with L-DOPA and DBS. - While L-DOPA increases beta power in the motor cortical regions, STN DBS acts differently by decreasing high beta power. - Both L-DOPA and DBS modulatory effects correlate with clinical scores. |
| Oscillatory and non-oscillatory features of  the magnetoencephalic sensorimotor  rhythm in Parkinson’s disease | Mikkel C. Vinding / Mikkel C. Vinding | 2024 | NPJ Parkinsons Dis | NatMEG, Department of Clinical Neuroscience, Karolinska Institutet, Stockholm, Sweden | PD=78  HC=60 | 6 | source | **Main point: Patients with PD show steeper 1/f decay and longer beta bursts, compared to controls. Beta bursts duration and rate are associated with clinical severity.**   - Resting state MEG data recorded from patients with PD and HC. The following Sensorimotor signal characteristics were evaluated in each group: alpha and beta power and central frequency, 1/f broadband features, and beta burst characteristics. - Patients with PD showed a steeper 1/f exponential slope and a steeper age-related decrease in the burst rate. - Out of all the above sensorimotor signal features, the burst rate was associated with increased bradykinesia severity, whereas burst duration was positively associated with axial symptoms. |
| Cortical Effects of Dopamine Replacement Account for Clinical  Response Variability in Parkinson’s Disease | Alex I. Wiesman / Alex I. Wiesman | 2025 | Mov Disord | Department of Biomedical  Physiology and Kinesiology, Simon Fraser University, 8888 University  Drive, Burnaby, BC V5A 1S6, Canada | PD=17  HC=20 | 5 | source | **Main point: Dopamine replacement therapy (DRT)-associated cortical beta power response occurs mainly in cortical regions rich in dopamine, and this response is associated with smaller clinical improvement from L-DOPA.**   - Resting state MEG data recorded in patients with PD before and after DRT administration. - DRT response maps were combined with cortical dopamine densities map. - Beta rhythmic response to DRT occurred more significantly in dopamine-rich cortical regions and was variable across participants. - Patients who exhibited strong beta response in dopamine-rich regions had a smaller clinical improvement from DRT. |
| Associations between neuromelanin  depletion and cortical rhythmic activity in  Parkinson’s disease | Alex I. Wiesman / Alex I. Wiesman | 2024 | Brain | Department of Biomedical Physiology & Kinesiology, Simon Fraser University, Burnaby, British Columbia, Canada  V5A 1S6 | PD=58  HC=65 | 5 | source | **Main point: Cortical alpha band is associated with the degree of locus coeruleus (LC) degeneration and with impaired attention scores.**   - Free task MEG combined with neuromelanin sensitive MRI was used to identify neurophysiological factors that are associated with the degeneration of dopaminergic and adrenergic cells in the substantia nigra (SN) and locus coeruleus (LC), respectively. - Cortical alpha band activity is increased in patients with decreased LC neuromelanin. This relationship is related to attention scores. - Cortical beta band activity is decreased in patients with decreased SN neuromelanin. |
| Subthalamic Nucleus Deep Brain Stimulation in the Beta  Frequency Range Boosts Cortical Beta Oscillations and  Slows Down Movement | Lucy M. Werner / Jan Hirschmann | 2025 | J Neurosci | Institute of Clinical Neuroscience and Medical Psychology, Medical Faculty and University Hospital Düsseldorf, Heinrich Heine University Düsseldorf,  Düsseldorf 40225, Germany | PD=14 | 5 | sensor | **Main point:** STN stimulation in the beta range reduces finger tap rate. The decrease in finger taps rate correlates with ipsilateral stimulation-induced beta power modulation.   - MEG recorded from 14 patients with PD during finger tapping, in two conditions: DBS off and DBS on with rate of 10-30 Hz (beta-band DBS). - Beta-band DBS reduced tap rate. Ipsilateral beta power increase correlated with tap decrease. |
| Analyzing Information Exchange in Parkinson's Disease via Eigenvector Centrality: A Source-Level Magnetoencephalography Study | Michele Ambrosanio / Pierpaolo Sorrentino | 2025 | Journal of Clinical Medicine | Institute of Applied Sciences and Intelligent Systems, National Research Council, 80078 Pozzuoli, Italy; | PD=47  HC=47 | 6 | source | **Main point: Significant differences were found between PD and HC in the corticocortical connectivity profile. Connectivity measures in PD correlated with clinical impairment.**   - Resting state MEG data was used to measure eigenvector centrality (EC, a measure of the importance of a node within a network)**.** - EC profiles differed between groups. In the PD group, frontal alpha EC correlated with clinical impairment. |
| Context-Dependent Modulations of Subthalamo-Cortical Synchronization during Rapid Reversals of Movement Direction in Parkinson's Disease | Lucie Winkler / Petra Fischer | 2025 | eLife | School of Physiology, Pharmacology & Neuroscience,  University of Bristol, Bristol, United Kingdom | PD=20 | 5 | source | **Main point: Movement start, stop, and reversal are associated with distinct modulation of beta power and coherence across motor nodes.**   - MEG and STN LFP were combined in 20 patients with PD to study dynamics during movement initiation, stop, and reversal. - Beta power and coherence are modulated differently during these movement conditions. - Movement reversal does not show a clear beta rebound (despite involving a brief stop in movement). |
| Alterations of Cortical Structure and Neurophysiology in Parkinson's Disease Are Aligned with Neurochemical Systems | Alex I. Wiesman / Alex I. Wiesman | 2024 | Annals of Neurology | McConnell Brain Imaging Centre, Montreal Neurological Institute, McGill University, Montreal, QC, Canada | PD=79  HC=65 | 6 | source | **Main point: Neurophysiological alterations in PD (compared to HC) occur primarily in brain regions rich in specific neurotransmitters/neurochemical systems, and the strength of this co-localization relates to motor deficits.**   - Task-free MEG from patients with PD and HC was used to examine neurophysiology co-localization with neurochemical systems. - Neurophysiological alterations were observed in regions rich in acetylcholinergic, serotonergic, and glutamatergic systems. This alignment was related to nonmotor deficits. - Cortical thinning, in contrast, occurs mainly in regions rich in noradrenergic systems. This alignment is related to motor deficits. |
| Early cortico-muscular coherence and cortical network changes in Parkinson's patients treated with MRgFUS | Elisa Visani / Silvana Franceschetti | 2024 | Frontiers in Neurology | Neurophysiopathology Unit,  Fondazione IRCCS Istituto Neurologico Carlo Besta, Milan, Italy | PD=16 | 5 | source | **Main point: Magnetic Resonance-guided Focused Ultrasound (MRgFUS) increases cortico-muscular**  **coherence (CMC) and decreases cortico-cortical connectivity.**   - MEG signals were evaluated in 16 patients with PD before and after MRgFUS during isometric hand extension. - Post-treatment, normalized beta. corticomuscular coherence (CMC) between the hand muscle and contralateral motor areas increased. - Post-treatment, beta corticocortical connectivity from the contralateral precentral region to other brain regions decreased. |
| The neurophysiological brain-fingerprint of Parkinson's disease | Jason da Silva Castanheira / Sylvain Baillet | 2024 | eBioMedicine | Montreal Neurological Institute, McGill University, Montreal, QC, Canada | PD=79  HC=10 | 5 | source | **Main point: Increased moment-to-moment neurophysiological variability in patients with PD contributes to a lower “ brain-fingerprint” based patient differentiation.**   - Resting state MEG recordings were used to test “brain-fingerprint” based individual differentiation accuracy, in both patients with PD and HC. - Overall, differentiation accuracy was lower for patients compared to controls. - The increased variability of arrhythmic brain components contributes to the lower differentiation accuracy in the PD group. - When isolating only the rhythmic spectral component, patients with PD showed lower variability compared to controls. |
| Low-beta versus high-beta band cortico-subcortical coherence in movement inhibition and expectation | Chunyan Cao / Chunyan Cao, Dianyou Li, and Bernadette C.M. van Wijk | 2024 | Neurobiology of Disease | Department of Neurosurgery, Center for Functional Neurosurgery, Ruijin Hospital, Shanghai Jiao Tong University School of Medicine, Shanghai, China | PD=20 | 6 | source | **Main point: Low beta and high beta subcortico-cortical coherence show different functional roles in facilitating and inhibiting movement.**   - STN/GPi-cortical coherence was evaluated in 20 patients with PD using LFP and MEG. - The Classical go/nogo task showed clear low beta coherence movement-related modulation (decrease followed by increase). - High beta coherence modulation in the same task was less clear and should be further investigated. |
| Adverse and compensatory neurophysiological slowing in Parkinson's disease | Wiesman, A. I. / Wiesman, A. I. and Baillet, S. | 2023 | Progress in Neurobiology | McConnell Brain Imaging Centre, Montreal Neurological Institute, McGill University, Montreal, Canada | PD=79  HC=65 | 5 | source | **Main point: Neurophysiological slowing is associated with clinical symptoms (motor and non-motor) along the sagittal gradient over the cortical anatomy.**   - Resting state MEG from 79 patients with PD shows that neurophysiological slowing is topographically associated with clinical symptoms. - Posterior slowing is associated with worse symptoms, while frontal slowing shows a compensatory role. |
| Aberrant neurophysiological signaling associated with speech impairments in Parkinson's disease | Wiesman, A. I. / Denise Klein or  Sylvain Baillet | 2023 | NPJ Parkinsons Dis | Montreal Neurological Institute, McGill University, Montreal, QC, Canada | PD=59  HC=65 | 5 | source | **Main point: Articulation impairments in patients with PD are associated with aberrant activity in the left inferior frontal cortex (LIFC), and functional connectivity of this region with somatomotor cortices mediates the influence of cognitive decline on speech deficits.**   - Speech impairment is significantly related to clinical features (UPDRS3, Hoehn and Yahr, MOCA). - Articulation impairment is uniquely associated with spectral pathology in the LIFC, with the greatest contribution from delta and alpha bands. - Patients with a weaker LIFC-somatomotor functional connectivity in the beta band exhibited worse articulation impairments. |
| Alterations of PAC-based resting state networks in Parkinson's disease are partially alleviated by levodopa medication | Sean Mertiens / Esther Florin | 2023 | Front Syst Neurosci | Institute of Clinical Neuroscience and Medical Psychology, Medical Faculty, Heinrich Heine University  Düsseldorf, Düsseldorf, Germany | PD=23  HC=24 | 5 | source | **Main point: Cortical PAC profile differs between patients with PD and HC. L-DOPA reduces this difference in the sensorimotor area.**   - Resting state MEG was recorded in 23 patients with PD and 24 HC. - PAC maps were calculated and compared between cohorts. - Significant changes were found between cohorts. L-DOPA administration in patients with PD led to network normalization. |
| Alterations of resting-state networks of Parkinson's disease patients after subthalamic DBS surgery | Matthias Sure / Matthias Sure, Esther Florin | 2023 | NeuroImage: Clinical | Institute of Clinical Neuroscience and Medical Psychology, Heinrich-Heine University, Düsseldorf, Germany | PD=27  HC=24 | 5 | source | **Main point: The stun effect (temporary improvement in motor symptoms induced by implantation of electrodes) exerts brain-wide changes in different functional resting state networks (RSN).**   - Analysis of the stun effect (motor improvement after electrode insertion) in 27 patients with PD. - RSN were identified in all conditions: sensory motor, visual, fronto-occipital, and frontal. - Each RSN was altered due to electrode implantation. - While the stun effect is associated with motor improvement, preoperative RSNs corresponded better with HC. |
| DBS-evoked cortical responses index optimal contact orientations and motor outcomes in Parkinson's disease | Rachel K. Spooner / Rachel K.  Spooner or Esther Florin | 2023 | NPJ Parkinsons Dis | Institute of Clinical Neuroscience and Medical Psychology, Heinrich-Heine University, Düsseldorf, Germany | PD=20 | 6 | sensor, source | **Main point: DBS-Evoked cortical response and quantitative movement outcomes can be used to characterize the optimal DBS contact orientation (=direction of current administration).**   - 24 patients with PD received monopolar STN-DBS stimulation during a standardized movement protocol with MEG recordings. - Directional specificity of STN-DBS current administration on accelerometer metrics and MEG data was interrogated. - Optimal contact orientations elicit larger DBS evoked cortical responses in the ipsilateral sensorimotor cortex. - Optimal contact orientations are differentially predictive of smoother movement profiles in a contact-dependent manner. |
| Cortical and Subcortical Changes in MEG Activity Reflect Parkinson's Progression over a Period of 7 Years | Lennard I. Boon / Lennard I. Boon | 2023 | Brain Topography | Department of Neurology, Amsterdam UMC, Vrije  Universiteit, Amsterdam, The Netherlands | PD=70  HC=21 | 7 | source | **Main point: Spectral measures can be used to characterize different stages of PD.**   - Repeated resting state MEG and clinical assessments were obtained in the context of a unique longitudinal study over a 7-year period. - Linear mixed models were used to analyze the relationship between neurophysiological (spectral power and functional connectivity) and clinical data.   Findings:   - At baseline, early-stage (drug naive) patients with PD demonstrated spectral slowing compared to healthy controls in both subcortical and cortical regions. - Over time, spectral slowing progressed in strong association with clinical measures of disease progression (cognitive and motor). - Global functional connectivity was not different between groups at baseline and hardly changed over time. |
| Cortex-wide topography of 1/f-exponent in Parkinson’s disease | Pascal Helson / [Pascal Helson](mailto:pashel@kth.se), [Mikkel C. Vinding](mailto:mikkelcv@drcmr.dk) or [Arvind Kumar](mailto:arvkumar@kth.se). | 2023 | NPJ Parkinsons Dis | School of Electrical Engineering and Computer Science, Science for Life Laboratory, KTH Royal Institute of Technology, Stockholm, Sweden | PD=17  HC=20 | 7 | source | **Main point: The spectral aperiodic component differs between PD and HC, across a wide range of cortical areas.**   - Resting state MEG was analyzed in patients with PD and HC. - The aperiodic component of the neural activity was quantified by fitting a power law k/f^(lambda). - The aperiodic component relationships to age and UPDRS3 were studied. - In all but frontal regions, lambda was significantly larger in PD than in HC. - Lambda was significantly correlated with age but not with UPDRS3. |
| Slowing of Frontal β Oscillations in Atypical Parkinsonism | Marius Krösche / Jan Hirschmann | 2023 | Movement Disorders | Institute of Clinical Neuroscience and Medical Psychology, Medical Faculty, Heinrich Heine University,  Düsseldorf, Germany | CBS=14  PSP=16  PD=33  HC=24 | 5 | source | **Main point: Atypical Parkinsonism (CBS,PSP) is associated with the slowing of beta oscillations, when compared to idiopathic PD.**   - Resting-state MEG in 14 CBS patients, 16 PSP patients, and 33 idiopathic PD patients. - Spectral power and frequency characteristics were compared between groups. - Atypical parkinsonism (CBS, PSP) showed a shift in beta peaks towards lower frequencies in frontal areas bilaterally. |
| Deep brain stimulation for Parkinson’s disease induces  spontaneous cortical hypersynchrony in extended motor  and cognitive networks | Maxwell B Wang / Maxwell B Wang | 2022 | Cerebral Cortex | Program of Neural Computation, Carnegie Mellon University, Pittsburgh, PA 15213, USA | PD=11  HC=34 | 5 | source | **Main point: DBS for PD induces cortical hypersynchrony across multiple brain areas.**   - Resting state cortical connectivity between HC, PD DBS-ON, and PD DBS-OFF. - DBS was found to increase high beta and gamma band synchrony in cortical circuits spanning the motor, occipitoparietal, middle temporal, and prefrontal cortices. - The above increase was when comparing DBS-ON to either control, or DBS-OFF. There was no significant change between DBS-OFF and controls. - Turning DBS-ON also increased network efficiency and strength, and subnetwork modularity in the high beta and gamma band, relative to DBS-OFF and controls. |
| Neural correlates of impaired response inhibition in the antisaccade task in Parkinson's disease | Josefine Waldthaler / Josefine Waldthaler | 2022 | Behavioural Brain Research | Department of Neurology, University Hospital Marburg, Marburg, Germany | PD=21  HC=21 | 7 | source | **Main point: Alterations in pre-stimulus prefrontal alpha and beta activity hinder proactive response inhibition and, in turn, result in higher error rates and prolonged response latencies in PD.**   - MEG data was recorded in 21 patients with PD and 21 HC. The cortical dynamics underlying response inhibition in the antisaccade task were explored.   Findings during the pre-stimulus preparatory period for antisaccades:   - Increase in beta band activity in the right dorsolateral prefrontal cortex (DLPFC) of HC (compared with prosaccades) that was not detectable in the PD group. - Less increase in alpha band power in bilateral FEF and reduced alpha band connectivity between the right DLPFC and right FEF in the PD group compared with HC (suggesting a reduced top-down control to inhibit pre-potent activation FEF in PD). - A positive correlation between the magnitude of pre-stimulus beta desynchronization in FEF and subsequent antisaccade latency in PD and HC (indicating a relationship between preparatory beta band modulation and effectiveness of subsequent antisaccade execution). |
| Functional connectivity maps of theta/alpha and beta coherence within the subthalamic nucleus region | Bernadette C.M. van Wijk / Bernadette C.M. van Wijk | 2022 | NeuroImage | Department of Human Movement Sciences, Faculty of Behavioural and Movement Sciences, Vrije Universiteit Amsterdam, the Netherlands | PD=21 | 6 | source | **Main point: Different locations within the STN have different cortical-STN coherence profiles.**   - Sites with theta-alpha coherence peaks were situated at significantly more inferior MNI coordinates than beta coherence peaks. - Sites with only theta/alpha coherence peaks (no beta) were mostly located near the border of sensorimotor and cognitive/associative subregions as defined by tractography based atlas of the STN. |
| Deep brain stimulation of subthalamic nucleus modulates cortical auditory processing in advanced Parkinson's Disease | Kati Valkonen / Hanna Renvall | 2022 | PLOS ONE | BioMag Laboratory, Helsinki University Hospital  Medical Imaging Center, Helsinki, Finland | PD=15 | 5 | source | **Main point: Long-term STN-DBS shows “normalization” of auditory evoked response, and thus may have long-lasting effects on cortical areas outside the motor regions.**   - patients with PD with STN-DBS were studied with MEG during auditory stimulation. - DBS resulted in significantly increased contra-ipsilateral auditory response latency difference at ~100 ms after stimulus onset compared to the preoperative state. - The effect is likely due to normalization of neuronal asynchrony in the auditory pathways. - These results indicate that STN-DBS in advanced PD patients has long-lasting effects on cortical areas outside those confined to motor processing. |
| Magnetoencephalography detects phase-amplitude coupling in Parkinson's disease | Masataka Tanaka / Takufumi Yanagisawa | 2022 | Scientific reports | Department of Neurosurgery, Osaka University Graduate School of Medicine,  Osaka, Japan | PD=23  HC=23 | 7 | source | **Main point: Beta-gamma phase amplitude coupling (PAC) in the resting state characterizes PD, and beta-gamma PAC in the sensorimotor cortices correlates with motor symptoms.**   - Resting state MEG was recorded in patients with PD and HC. - Power spectra and coupling between gamma and alpha/beta were compared between groups. - Patients with PD showed significant beta-gamma PAC that was widely distributed in the sensorimotor, occipital, and temporal cortices. - Beta and gamma band power differed significantly between participants in the two groups. - Beta-gamma PAC in the sensorimotor cortices correlated significantly with motor symptoms of PD. |
| Cortical network formation based on subthalamic beta bursts in Parkinson's disease | Matthias Sure / Matthias Sure, Esther Florin | 2022 | NeuroImage | Institute of Clinical Neuroscience and Medical Psychology, Medical Faculty, Heinrich-Heine University Düsseldorf, Düsseldorf, Germany | PD=26 | 5 | source | **Main point: STN beta bursts are involved in network formation between the STN and the cortex.**   - Event-related magnetic fields (ERFs) were averaged time-locked to STN beta bursts. - ERF exhibiting activity significantly different from baseline activity were localized within areas functionally related to associative, limbic, motor systems, and areas pertinent for visual and language processing. |
| Profiling Parkinson's disease cognitive phenotypes via resting-state magnetoencephalography | Olivier B. Simon / Isabelle Buard | 2022 | J Neurophysiol | University of Colorado Denver, Aurora,  Colorado | PD=89 | 6 | source | **Main point: In the spectrum of normal cognition, mild cognitive impairment (MCI), and Parkinson’s disease dementia (PDD),most dramatic changes occur in the transition from normal cognition to MCI.**   - A resting state MEG cross-sectional study of 89 persons with PD, divided into 3 groups. - Groups in the study: normal cognition, MCI, PDD. - Higher power in lower frequency bands (delta and theta) is observed along with more severe cognitive impairment. - Widespread whole brain group differences were found in the beta band, with significant changes mostly occurring between the normal cognition and MCI groups. |
| Single-Trajectory Multiple-Target Deep Brain Stimulation for Parkinsonian Mobility and Cognition | Sanskriti Sasikumar / Alfonso Fasano | 2022 | Movement Disorders | Division of Neurology, University of Toronto, Toronto, Ontario,  Canada | PD=6 | 7 | source | **Main point: DBS of nucleus basalis of meynert (NBM) changes brain signal characteristics (power, connectivity), but does not improve cognition.**   - Compared to NBM OFF, the NBM ON condition showed increased low frequency (delta, and or theta) activity in the left frontal, parietal, and temporal lobe regions and increased high frequency (beta and or low gamma) activity more posteriorly – including right occipital and cerebellar regions. - NBM increased coherence within the left hemisphere (both intra- and inter-hemispherically). - NBM DBS did not improve cognition. |
| Brain Networks and Cognitive Impairment in Parkinson's Disease | Rosaria Rucco / Giuseppe Sorrentino | 2022 | Brain Connectivity | Department of Motor Sciences and Wellness, University of Naples Parthenope, Naples, Italy | PD-CI=20  PD-NC=19  HS=20 | 7 | source | **Main point: Large-scale rearrangements occur selectively in cognitively compromised PD patients and are correlated to cognitive impairment.**   - Resting state MEG for three groups: patients with cognitive impairment (PD-CI), patients without cognitive impairment (PD-NC), and healthy subjects (HS). - Functional connectivity (PLM, graph analysis) was measured in each group. - Reduced global and nodal PLM in several temporal, parietal, and occipital areas within the left hemisphere (in the gamma band) in PD-CI (compared to the other two groups). - PD-CI patients also showed differences in global topological features across multiple frequency bands. - There was a statistically significant correlation between MOCA and some global topological features. |
| Cortical beta burst dynamics are altered in Parkinson's disease but normalized by deep brain stimulation | K. Amande M. Pauls / K. Amande M. Pauls | 2022 | NeuroImage | BioMag Laboratory, HUS Medical imaging Center, Helsinki University Hospital, Helsinki, Finland | PD=16  HC=21 | 5 | sensor | **Main point: Beta bursts characteristics are affected in PD, and STN-DBS normalizes some of these changes to resemble healthy control beta bursts activity.**   - Resting state MEG recordings were used to study sensorimotor cortical beta bursts. - Groups in the study: patients with PD (+/- STN-DBS) and HC. - Changes in beta bursting amplitude and duration typical of PD can be observed in the sensorimotor cortex. - In untreated patients with PD: bursts are more clustered, and re-burst probability was higher for longer compared to shorter bursts. - DBS stimulation “normalizes” bursts characteristics to resemble HC. |
| Conflict Detection in a Sequential Decision Task Is Associated with Increased Cortico-Subthalamic Coherence and Prolonged Subthalamic Oscillatory Response in the β Band | E. Zita Patai / Rafal Bogacz | 2022 | The Journal of Neuroscience | Medical Research Council Brain Network Dynamics Unit, Oxford University, Oxford OX1 3TH, United Kingdom | PD=13  HC=13 | 5 | sensor, source | **Main point: Judgement tasks performed by patients with PD show distinct neural markers for conflicted cues. Behaviorally, patients with PD perform worse than HC.**   - Patients with PD and HC performed a judgement task (cues and then decision making).   Key findings:   - Prolonged beta oscillations in the STN, with concurrent increase in communication with the frontal cortex, are observed when presented with conflict information. - Behavior profile: reduced tendency to respond after conflict, as well as suboptimal cue integration in patients with PD. |
| Neuronal oscillations predict deep brain stimulation outcome in  Parkinson's disease | Jan Hirschmann / Jan Hirschmann | 2022 | Brain Stimulation | Institute of Clinical Neuroscience and Medical Psychology, Medical Faculty, Heinrich Heine University, Düsseldorf, Germany | PD=36 | 5 | source | **Main point: Neural oscillations are predictive of DBS outcome.**   - Objective – to predict motor symptom improvement from subthalamic power and subthalamo-cortical coherence. - Machine learning techniques to simultaneously record magnetoencephalography and LFP data. - A few features sufficed to make accurate predictions. - Coherence comprised more information in fewer features than subthalamic power (reaching a correlation between actual and predicted outcomes > 0.8). - Most important local feature: subthalamic high beta power. - Most important connectivity features: subthalamo-parietal coherence in the very high frequency band (>200 Hz), and subthalamo-parietal coherence in the low gamma band (36-60 Hz). |
| Cortical oscillatory dysfunction in Parkinson  disease during movement activation and  inhibition | Elizabeth A. Disbrow / Elizabeth A. Disbrow | 2022 | PLOS ONE | LSU Health Shreveport Center for Brain Health, Shreveport, Louisiana, United States of America | PD=18  HC=18 | 7 | source | **Main point: Description of PD associated cortical power changes during movement activation and inhibition.**   - Participants performed a cue/target task that required initiation of un-cued movement (activation) or inhibition of cued movement. - MEG recordings were used to examine event related changes in oscillatory power amplitude, peak latency and frequency in cortical networks subserving response activation and inhibition. - PD related changes in power and latency were noted most frequently in the beta band # abnormal power and delayed peak latency in the alpha band in pre-SMA was suggestive of compensatory mechanism. - PD peak power was delayed in the pre SMA, motor cortex, medial frontal gyrus only for activation, which is consistent with deficits in un-cued movement initiation characteristic id PD. |
| Reduced cortico-muscular beta coupling in Parkinson’s disease predicts motor impairment | Nahid Zokaei / Nahid Zokaei | 2021 | BRAIN COMMUNICATIONS | Oxford Centre for Human Brain Activity (OHBA), Wellcome Centre for Integrative Neuroimaging, Department of Psychiatry,  University of Oxford, UK | PD=17  HC=17 | 6 | sensor | **Main point: Reduced beta cortico-muscular coherence in** **patients with PD is associated with motor impairment.**   - Cortico-muscular coherence (CMC) was examined in patients with PD and HC (equating grip strength between groups). - PD group showed a marked reduction in beta CMC. - The reduced CMC was related to motor symptoms (individuals with lower CMC showed worse motor symptoms). |
| Flexible brain dynamics underpins complex behaviours as observed in Parkinson's disease | Pierpaolo Sorrentino / Pierpaolo Sorrentino | 2021 | Sci Rep | Department of Engineering, University of Naples Parthenope, Centro Direzionale,Naples,  Italy | PD=39  HC=38 | 7 | source | **Main point: Symptoms in PD relate to impaired brain flexibility; this impairment preferentially involves the BG, and beta band hyper synchronization is associated with reduced brain flexibility.**   - The functional repertoire (= the number of distinct configurations of neural activity) was studied using MEG. - Stereotyped brain dynamics and reduced flexibility were found in PD. - The intensity of flexibility reduction was proportional to symptom severity, which can be explained by beta band hyper synchronization. - Basal ganglia were prominently involved in the abnormal patterns of brain activity. |
| A novel approach to understanding Parkinsonian cognitive decline using minimum spanning trees, edge cutting, and magnetoencephalography | Olivier B. Simon / Debashis Ghosh | 2021 | Sci Rep | Department of Biostatistics and Informatics, Colorado School of Public Health, University of Colorado Anschutz  Medical Campus, Aurora, CO, USA | PD=89 | 7 | source | **Main point: Minimum spanning tree (MST) can be used to quantify/predict/diagnose PD dementia.**   - MST was calculated for 89 patients with PD from resting state MEG. - MST was used to relate the severity of Parkinsonian cognitive impairment to neural connectivity changes. - Altered connectivity in Parkinsonian dementia was observed. |
| Differential dopaminergic modulation of spontaneous cortico-subthalamic activity in Parkinson's disease | Abhinav Sharma / Abhinav Sharma and Esther Florin | 2021 | Elife | Institute of Clinical Neuroscience and Medical Psychology, Medical Faculty,  Heinrich-Heine University Du¨sseldorf, Du¨sseldorf, Germany | PD=17 | 6 | source | **Main point: Dopaminergic medications have a modulatory effect on brain networks.**   - The effect of dopaminergic medication was characterized by employing Markov chain analysis on LFP and MEG data. - Dopaminergic medication led to coherence within the medial and orbitofrontal cortex in the delta/theta frequency range. - Dopamine caused the beta band activity to switch from STN-mediated motor network to the frontoparietal-mediated one. |
| Individual Magnetoencephalography Response Profiles to Short-Duration L-Dopa in Parkinson's Disease | Edgar Peña / Jawad A. Bajwa | 2021 | Front Hum Neurosci | Department of Neurology, National Neuroscience  Institute, King Fahad Medical City, Riyadh, Saudi Arabia | PD=20 | 6 | source | **Main point: Subject-specific machine learning approaches based on neuroimaging provide tools to predict outcomes in therapies for PD.**   - Changes in UPDRS3 following short-duration L-DOPA response were characterized. - MEG data was collected. - Linear support vector machines were used to quantify motor cortical oscillatory frequency profiles that best predicted medication state. |
| Neural signatures of hyperdirect pathway activity in Parkinson's disease | Ashwini Oswal / Ashwini Oswal, Peter Brown and Vladimir Litvak | 2021 | Nature Communications | MRC Brain Network Dynamics Unit, University of Oxford, Oxford, UK | PD=32 | 7 | source | **Main point: The hyper-direct pathway in PD has a pathophysiological role and a spectral signature.**   - Recordings from: human STN, GPi, MEG, and tractography imaging. - Coherence between SMA and STN within high beta (21-30 Hz) but not low beta (13-21 Hz) range correlated with “hyper-direct pathway” fibre density between these structures. - SMA drove STN activity selectively at high beta frequencies, suggesting that high beta frequencies propagate from the cortex to the BG via the hyper-direct pathway. - Computational modelling revealed that exaggerated high beta hyper-direct pathway activity can provoke the generation of widespread pathological synchrony at lower beta frequencies. |
| Altered neural oscillations during complex sequential movements in patients with Parkinson's disease | Marie C. McCusker / Elizabeth Heinrichs-Graham | 2021 | Neuroimage Clin | Institute for Human Neuroscience, Boys Town National Research Hospital, Boys Town, NE, USA | PD=23  HC=27 | 5 | Sensor, source | **Main point: patients with PD show altered neural oscillations during complex sequential movements.**   - Participants performed simple and complex finger tapping sequences with MEG recordings. - patients with PD were slower at executing sequences. - Patients with PD also exhibited weaker beta complexity-related effects in the right medial frontal gyrus and weaker complexity-related alpha activity in the right posterior and inferior lobules. |
| Functional connectivity of spoken language processing in early-stage Parkinson's disease: An MEG study | Rasha Hyder / Rasha Hyder and Yury Shtyrov | 2021 | Neuroimage Clin | Center of Functionally Integrative Neuroscience (CFIN), Department of Clinical Medicine, Aarhus University, Aarhus, Denmark | PD=17  HC=15 | 5 | source | **Main point: Neurophysiological response to spoken word stimuli can be used to differentiate patients with PD from HC.**   - MEG recordings from a group of newly diagnosed patients with PD and age-matched HC. Both groups were passively presented with spoken word stimuli. - logistic regression classifier was used to classify participants as either patients with PD or healthy controls based on functional connectivity within the temporo-fronto-parietal cortical language networks. - Classification accuracy reached 0.781, p=0.003. |
| Expiratory Muscle Strength Training for Therapy of Pharyngeal Dysphagia in Parkinson's Disease | Inga Claus / Inga Claus | 2021 | Movement Disorders | Department of Neurology with Institute of Translational Neurology, University Hospital of Muenster, Muenster, Germany | PD=50 | 8 | source | **Main point: Expiratory muscle strength training may significantly reduce dysphagia severity in patients with PD.**   - In a double-blind, randomized, controlled trial, 50 patients with hypokinetic pharyngeal dysphagia performed a 4-week expiratory muscle strength training (25 active devices, 25 sham devices). - Swallowing function was evaluated before and after a training period. - The active group showed significant improvement in flexible endoscopic evaluation after the training period. - In addition, the effect of training on cortical activity during swallowing was assessed with MEG in 22 participants (active:sham 11:11). - No statistically significant differences were found in MEG analysis. |
| Structural and functional correlates of subthalamic deep brain stimulation-induced apathy in Parkinson's disease | Lennard I. Boon / Lennard I. Boon | 2021 | Brain Stimul | Amsterdam UMC, Vrije Universiteit Amsterdam, Neurology, Amsterdam Neuroscience, Amsterdam, the Netherlands | PD=26 | 6 | source | **Main point: Apathy after STN-DBS is not necessarily related to dose reductions of dopaminergic medication but may be an effect of the stimulation itself.**   - In patients with PD, apathy scores were acquired before and 6 months (or more) after STN-DBS. - MEG recordings were performed 6 months (or more) after DBS placement.   Analysis showed:   - Significant increase in apathy after DBS. - Change in apathy score did not correlate with improvement in motor function or reduction of dopaminergic medication. - Increase in apathy severity correlated with decrease in alpha1 functional connectivity of the dorsolateral prefrontal cortex. - For the left hemisphere, increase in apathy was associated with a more dorsolateral stimulation location. |
| Reduction of spontaneous cortical beta bursts in Parkinson's disease is linked to symptom severity | Mikkel C. Vinding / Mikkel C. Vinding | 2020 | Brain Commun | Department of Clinical Neuroscience, NatMEG, Karolinska Institutet, Sweden | PD=19  HC=19 | 7 | source | **Main point: Cortical beta bursts in PD are characterized by a lower rate compared to HC. Lower rate burst is linked to increased symptom severity.**   - Time course beta band activity in the sensorimotor cortex from MEG data. Then, beta bursts’ characteristics were extracted. - patients with PD (OFF med) had a lower burst rate compared to controls. - Lower beta burst rate is related to increased severity in bradykinesia and tremor. |
| Identification of nonlinear features in cortical and subcortical signals of Parkinson's Disease patients via a novel efficient measure | Tolga Esat Özkurt / Tolga Esat Özkurt | 2020 | Neuroimage | Wellcome Trust Centre for Neuroimaging, UCL Institute of Neurology, London, UK | PD=14 | 5 | source | **Main point: Particular frequency bands and brain regions display nonlinear features closely associated with distinct motor symptoms and functions in PD.**   - “Nonlinearity” was measured in the time signal in both MEG and LFP recorded data. - Nonlinearity in subcortical high beta and cortical alpha was observed. - **Subcortical nonlinearity** was seen in the OFF-med state; the degree of nonlinearity was correlated with contralateral tremor severity. - **Cortical nonlinearity** was seen in the ON medication state; the degree of nonlinearity was correlated with contralateral akinesia and rigidity. |
| Dissecting beta-state changes during timed movement preparation in Parkinson's disease | Simone G. Heideman / Anna C. Nobre | 2020 | Prog Neurobiol | Oxford Centre for Human Brain Activity, Wellcome Centre for Integrative Neuroimaging, Department of Psychiatry, University of Oxford, Oxford, United Kingdom | PD=18  HC=18 | 5 | sensor | **Main point: Hidden Markov model analysis of MEG data revealed that increased gaps between beta “bursts” are associated with decreased beta power during** **movement planning. Also, this process can uniquely describe the impairment in movement preparation in patients with PD.**   - MEG was used in patients with PD and HC to investigate beta-event dynamics during timed movement preparation. - Using hidden Markov chain analysis, beta state interval time (=increase gap length between “bursts”) explained the overall decrease in beta power during timed movement preparation and uniquely captured the impairment in such preparation in patients with PD. |
| Resting state activity and connectivity of the nucleus basalis of Meynert and globus pallidus in Lewy body dementia and Parkinson's disease dementia | James Gratwicke / Vladimir Litvak | 2020 | Neuroimage | Wellcome Centre for Human Neuroimaging, UCL Institute of Neurology, 12 Queen Square, London, UK | PDD=6  DLB=5 | 5 | source | **Main point: Parkinson’s disease dementia (PDD) and dementia with Lewy bodies (DLB) seems to be distinguishable from a neurophysiological perspective**.   - MEG and bilateral LFP from nucleus basalis of Meynert (NBM) and internal globus pallidus (GPi). Recording from 6 PDD patients and 5 DLB patients. - **Similarities between PDD and DLB:** spectral peak in theta band in both GPi and NBM; Similar spatial and spectral patterns of coupling with cortex (theta band network linking NBM/GPi to temporal cortical regions, and also beta band network coupling NBM/GPi to sensorimotor areas). - **Differences between PDD and DLB:** oscillatory power in the low beta (13-22 Hz) band was significantly higher in the GPi in PDD patients compared to DLB; also, coherence in the high beta (22-35 Hz) band between GP and lateral sensorimotor cortex was significantly higher in DLB compared to PDD. |
| L-dopa treatment increases oscillatory power in the motor cortex of Parkinson's disease patients | Chunyan Cao / Bomin Sun, Vladimir Litvak | 2020 | Neuroimage Clin | Department of Neurosurgery, Aﬃliated Ruijin Hospital, Shanghai JiaoTong University School of Medicine, Shanghai, China | PD=21 | 7 | source | **Main point: L-DOPA treatment increases oscillatory power in the motor cortex of patients with PD, and this increase is significantly correlated with motor improvement**.   - Patients with PD were recorded with MEG in L-DOPA ON and OFF states. - A significant correlation between the magnitude of L-DOPA-induced 18-30 Hz motor cortical power increase and the degree of improvement in contralateral akinesia and rigidity was found. - Power in the same range was also inversely correlated with the combined akinesia and rigidity score in the OFF state, but not in the ON state. |
| Motor effects of deep brain stimulation correlate with increased functional connectivity in Parkinson's disease: An MEG study | Lennard I. Boon / Lennard I. Boon | 2020 | Neuroimage Clin | Amsterdam UMC, Vrije Universiteit Amsterdam, Neurology, Amsterdam Neuroscience, Amsterdam, the Netherlands | PD=18 | 6 | source | **Main point: STN-DBS has a distributed effect on the resting state brain; improvement in bradykinesia/rigidity may be mediated by an increase in alpha2 (10-13 Hz) and low beta functional connectivity.**   - Resting state MEG recording and assessment on motor function were obtained from 18 patients with PD with bilateral STN-DBS, on and off stimulation. - For each brain region, source space spectral power and functional connectivity were estimated.   Stimulation effect:   - Increase in average peak frequency and suppression of absolute band power (for delta to low beta band) in the sensorimotor cortices. - Significant changes (decreases and increases) in low beta band functional connectivity. - Improvement in bradykinesia/rigidity was significantly related to an increase in alpha 2 and low beta functional connectivity (across multiple areas). |
| Deep Brain Stimulation Does Not Modulate Auditory-Motor Integration of Speech in Parkinson's Disease | Bahne H. Bahners / Bahne H. Bahners | 2020 | Front Neurol | Institute of Clinical Neuroscience and Medical Psychology, Medical Faculty, Heinrich Heine University Düsseldorf,  Düsseldorf, Germany | PD=20 | 6 | source | **Main point: Subthalamic DBS appears to have no substantial effect on vocal compensation.**   - 20 patients with PD with STN-DBS were exposed to pitched-shifted acoustic feedback during vowel vocalization and subsequent listening. - Voice and brain activity were measured on and off DBS stimulation, using MEG. - Vocal responses and auditory evoked responses time locked to the onset of pitch-shifted feedback were examined. - No differences of vocal responses to pitch-shifted feedback between the stimulation conditions were found. |
| Attenuated NoGo-related beta desynchronisation and synchronisation in Parkinson's disease revealed by magnetoencephalographic recording | Hung-Ming Wu and Fu-Jung Hsiao / Yung-Yang Lin | 2019 | Sci Rep | Institute of Brain Science, National Yang-Ming University, Taipei, Taiwan | PD=12  HC=13 | 7 | source | **Main point: Event-related desynchronization/synchronization (ERD, ERS) characteristics in Go/NoGo** **test differ between PD and HC groups, with a significant link to motor symptoms.**   - MEG recording during Go/NoGo task in patients with PD   And HC.  Compared to HC, patients with PD had a:   - Significant reduction in beta ERD during NoGo conditions. - Significant reduction in beta ERS during both Go and NoGo conditions. - Delayed onset of ERD and ERS in in Go conditions. - NoGo ERS was negatively correlated with UPDRS3 scores in patients with PD. |
| Attenuated beta rebound to proprioceptive afferent feedback in Parkinson's disease | Mikkel C. Vinding / Mikkel C. Vinding | 2019 | Sci Rep | natMeG, Department of clinical neuroscience, Karolinska institutet, Stockholm, Sweden | PD=12  HC=16 | 7 | sensor | **Main point: Patients with PD show attenuated beta rebound to proprioceptive stimulation (passive movement), when compared to HC.**   - Cortical oscillations (MEG) in the mu/beta band (8-30 Hz) in the processing of proprioceptive stimulation were examined in patients with PD and HC. - Beta rebound after the passive movement was almost absent in patients with PD compared to HC. - No difference in the degree of beta rebound attenuation was found between patients ON and OFF levodopa medication. |
| Pre-stimulus beta power modulation during motor sequence learning is reduced in 'Parkinson's disease | Sarah Nadine Meissner / Sarah Nadine Meissner | 2019 | Neuroimage Clin | Institute of Clinical Neuroscience and Medical Psychology, Medical Faculty, Heinrich-Heine-University Dusseldorf, Dusseldorf, Germany | PD=20  HC=20 | 5 | sensor | **Main point: Pre-stimulus beta power suppression is reduced in PD compared to HC. Behaviorally, patients with PD show reduced motor** **sequence learning capability, compared to HC.**   - Patients with PD and HC performed a serial reaction time task (SRTT) in which reaction time gain presumably reflects the ability to anticipate subsequent sequence items. - Neuromagnetic activity was recorded using MEG.   Patients with PD vs HC:   - Patients with PD exhibited a small reaction time gain in sequence relative to random control trials (indicating reduced learning). - Patients with PD showed reduced pre-stimulus beta power suppression. |
| Aberrant resting-state oscillatory brain activity in Parkinson's disease patients with visual hallucinations: An MEG source-space study | M. Dauwan / M. Dauwan | 2019 | Neuroimage Clin | Neuroimaging Center, University Medical Center Groningen, University of Groningen, Groningen, the  Netherlands | PD Hall+=20  PD Hall-= 20 | 6 | source | **Main point: Compared to patients with no hallucination or multimodal hallucinations, patients with only visual hallucinations showed slowing of MEG-based resting state brain activity.**   - Resting state MEG were obtained from 20 patients with PD with hallucinations (Hall+), and 20 patients with PD without hallucinations (Hall-). - Hall+ was subdivided into 10 patients with visual hallucinations only (VH), and 10 patients with multimodal hallucinations. - Peak frequency and relative power in six frequency bands were compared.   Compared to Hall- group, unimodal Hall+ patients showed:   - Significantly higher relative power in the theta band. - Significantly lower relative power in the beta and gamma bands. - Lower peak frequency. - Compared to unimodal Hall+, multimodal Hall+ showed: - Significantly higher peak frequency. |
| Levodopa Reduces the Phase lag Index of Parkinson's Disease Patients: A Magnetoencephalographic Study | Chunyan Cao / Dianyou Li | 2019 | Clinical Eeg and Neuroscience | Department of Functional Neurosurgery, Ruijin Hospital, Afﬁliated to  Shanghai JiaoTong University School of Medicine, Shanghai, China | PD=18  HC=18 | 7 | sensor | **Main point: Patients with PD showed a higher mu PLI in the sensorimotor area, relative to HC. The improvement in motor symptoms of patients with PD by levodopa was correlated to the inhibition of beta PLI in the sensorimotor area.**   - 18 patients with PD were measured by MEG in the levodopa OFF and ON states and compared to HC. - Averaged PLI was measured in 4 MEG sensor regions (frontal, temporal, parietal, occipital).   Compared to HC:   - Alpha PLI in the frontal and parietal areas was elevated in patients with PD. - The above elevation was reversed by levodopa treatment. - The alternations in UPDRS3 total scale and changes in akinesia scale were correlated with the change of beta PLI in the parietal area. - There was a negative correlation between the age of the PD patient and the change in alpha PLI in the left frontal area. |

PD, Parkinson’s Disease

HC, Healthy Controls

MEG, Magnetoencephalography

L-DOPA, levodopa

DBS, Deep Brain Stimulation

STN, Subthalamic Nucleus

LFP, Local Field Potentials

GP, Globus Pallidus

GPi, Globus Pallidus Internus

UPDRS3, Unified Parkinson’s Disease Rating Scale Part-III

PAC, Phase-amplitude Coupling

CBS, Corticobasal Syndrome

PSP, Progressive Supranuclear Palsy

FEF, Frontal Eye Field

MNI, Montreal Neurological Institute

PLM, Phase Linearity Measurement

MoCA, Montreal Cognitive Assessment

SMA, Supplementary motor area

BG, Basal Ganglia

PLI, Phase Lag Index
